# Supplementary material for: IFNα induces CCR5 in CD4+ T cells of HIV patients causing pathogenic elevation
Source: Commun Med (Lond). 2024 Mar 19;4:52. doi: 10.1038/s43856-024-00453-7 (PMC10951336; doi:10.1038/s43856-024-00453-7)
Supplement: Supplementary file 1 — Supplementary Information [file 43856_2024_453_MOESM1_ESM.pdf]

Supplementary Tables and Figures:

Supplementary Table 1: Clinical data for HIV patients

Table 1a : EC patients

**EC**	HIV dx	Age	Sex	Sample date	CD4 at sample date	VL	HLA B57	Race	HIV risk factor	Treatment at the time of sampling
3	1990-2000	60-80	M	10/12/2012	1140	334	-	AA	IDU	None
4	1990-2000	40-60	F	22/08/2011	952	<40	+	AA	HS	None
6	1990-2000	40-60	F	31/05/2011	1889	<75	+	AA	IDU	None
8	1980-1990	40-60	M	01/02/2010	1018	<40	+	AA	IDU	None
9	2000-2010	40-60	M	25/09/2014	496	<40	+	AA	IDU	None
11	1990-2000	60-80	M	10/72/2011	917	<40	+	AA	IDU	None
13	1990-2000	40-60	M	21/02/2012	864	<40	-	AA	IDU	None
31	1990-2000	40-60	M	23/05/2012	587	<48	-	AA	MSM	None
32	1990-2000	40-60	F	23/05/2012	643	<48	-	AA	IDU	None
42	1990-2000	60-80	M	13/11/2013	731	<220	+	AA	IDU	None
47	2000-2010	40-60	F	01/12/2013	891	<20	-	AA	IDU	None
51	2000-2010	40-60	F	20/10/2014	1250	<40	+	AA	HS	None
52	1990-2000	40-60	M	01/08/2012	340	<20	+	AA	IDU	None
55	1990-2000	40-60	M	01/02/2013	482	50	-	AA	IDU	None
58	1990-2000	40-60	F	01/03/2013	1745	59	-	AA	HS	None
63	1980-1990	40-60	F	24/01/2014	632	32	+	AA	IDU	None
65	1990-2000	40-60	M	01/06/2013	1792	<48	+	AA	HS	None
68	2000-2010	40-60	F	01/07/2010	584	169	+	AA	HS	None

Dx : Diagnosis; M : Male; F : Female; AA : Afro American ; IDU : Injection Drug Use ; HS : Homosexual MSM : Men who have sex with men

Table 1b: HIV untreated patients

Untreated patients study_ID	Age	Sex	sample date	CD4 at sample date	VL	Treatment at the time of sampling	Origin
UP 1	NA	NA	03/11/2004	506	23808	None	NIH (Bethesda)
UP 2	NA	NA	22/04/2004	516	58059	None	
UP 3	NA	NA	01/10/2005	230	153719	None	
UP 4	NA	NA	27/09/2005	327	132773	None	
UP 5	NA	NA	19/06/2006	419	1715	None	
UP 6	NA	NA	04/10/2007	513	12015	None	
UP 7	NA	NA	13/08/2007	447	21701	None	
UP 8	NA	NA	13/03/2008	319	4785	None	
UP 9	NA	NA	05/11/2011	756	7011	None	
UP 10	NA	NA	08/10/2005	445	407	None	
UP 11	NA	NA	11/10/2009	292	3012	None	
UP 12	NA	NA	10/07/2010	477	29257	None	
UP 13	NA	NA	07/22/08	639	<50	None	
UP 14	NA	NA	11/17/2008	267	26059	None	
UP 15	NA	NA	03/23/2006	470	5384	None	
UP 16	NA	NA	11/07/2012	740	11417	None	
UP 17	NA	NA	01/28/2014	344	63954	None	
UP 18	NA	NA	11/04/2010	872	7180	None	
UP 19	NA	NA	12/03/2002	196	17809	None	
UP 20	20-40	F	10/26/2020	470	2480	None	Laboratoire de Référence SIDA (Liège)
UP 21	20-40	M	12/03/2020	100	24800	None	
UP 22	20-40	M	12/03/2020	139	32800	None	
UP 23	20-40	F	12/03/2020	356	9790	None	
UP 24	20-40	M	12/08/2020	390	588000	A few days	
UP 25	20-40	M	12/09/2020	1064	14700	None	
UP 26	20-40	M	12/14/2020	30	850000	None	
UP 27	40-60	M	02/24/2021	20	824	A few days	
UP 28	40-60	F	03/05/2021	280	82700	None	
UP 29	20-40	M	03/16/2021	577	157000	None	
UP 30	20-40	F	03/19/2021	180	73700	None	
UP 31	40-60	M	01/21/2022	321	122000	None	
UP 32	40-60	M	02/18/2022	489	40000	None	
UP 33	40-60	M	02/25/2022	449	1420000	None	
UP 34	40-60	M	12/17/2021	405	49800	None	
UP 35	20-40	M	12/30/2021	260	80900	None	
UP 36	40-60	M	07/20/2021	739	20300	None	

NA : Non Available

Table 1c: HIV treated patients

Treated patients study ID	Age	Sex	sample date	CD4 at sample date	VL	Origin
TP 1	NA	NA	02/10/2017	685	<40	NIH (Bethesda)
TP 2	NA	NA	1/19/2017	912	<40	
TP 3	NA	NA	11/27/2017	NA	na	
TP 4	NA	NA	12/08/2016	744	<40	
TP 5	NA	NA	7/19/2018	NA	<40	
TP 6	NA	NA	4/13/2018	NA	<40	
TP 7	NA	NA	9/26/2018	NA	<40	
TP 8	NA	NA	03/07/2012	417	<40	
TP 9	NA	NA	3/27/2017	809	<40	
TP 10	NA	NA	08/10/2006	503	<50	
TP 11	NA	NA	05/09/2012	402	<50	
TP 12	NA	NA	04/02/2013	627	<40	
TP 13	NA	NA	04/30/2010	603	<50	
TP 14	NA	NA	08/25/2010	404	<50	
TP 15	NA	NA	10/15/10	459	<50	
TP 16	NA	NA	10/21/2014	1328	<40	
TP 17	NA	NA	05/13/2016	410	<40	
TP 18	NA	NA	08/06/2010	682	<40	
TP 19	NA	NA	08/19/2004	437	<50	
TP 20	40-60	M	04/12/2021	712	<20	Laboratoire de Référence SIDA (Liège)
TP 21	40-60	M	04/12/2021	1075	<20	
TP 22	20-40	M	04/22/2021	645	<20	
TP 23	40-60	M	10/27/2020	869	<20	
TP 24	40-60	M	07/13/2021	1016	<20	
TP 25	40-60	M	07/13/2021	788	<20	
TP 26	40-60	F	07/14/2021	960	<20	
TP 27	20-40	M	07/28/2021	1530	<20	

NA : Non Available

Supplementary Table 2: mAb list for immune cells panel.

	Markers	Fluorochrome	Clone	Origin	Dilution
**Immune cell types**	CD3	AF532	UCHT1	Invitrogen	1/20
	CD4	BV510	RPA-T4	Biolegend	1/40
	CD8	BV750	RPA-T8	Biolegend	1/300
	CD56	BV711	HCD56	Biolegend	1/150
	CD16	Ef450	eBioCB163	Invitrogen	1/600
	TCR-γδ	BV480	11F2	BD Biosciences	1/40
	CD19	BV750	HIB19	Biolegend	1/40
	CD14	AF647	MOP9	BD Biosciences	1/300
	CD123	PerCpCy5.5	7G3	BD Pharmingen	1/10
	CD11c	BV605	3.9	Biolegend	1/20
**Immune Activation / Maturation**	CD45RA	FITC	REA562	Miltenyi Biotech	1/400
	CCR7	BV421	G043H7	Biolegend	1/40
	CD28	APC-R700	CD28.2	BD Biosciences	1/80
	CD25	BV786	M-A251	BD Biosciences	1/40
	HLA-DR	APCCy7	1243	Biolegend	1/1000
	CD26	PE	BA5b	Biolegend	1/300
	CD39	PeCy7	A1	Biolegend	1/300
	CD38	PerCPeF710	HB7	Invitrogen	1/40
**Immune checkpoint**	PD1	BV650	EH12.2H7	Biolegend	1/40
	CTLA-4	PeCy5	BNI3	BD Biosciences	1/20
	KIR2DL1	APC	REA284	Miltenyi Biotech	1/100
	KIR3DL1/DL2	APC	REA970	Miltenyi Biotech	1/100
	KIR2DL2/DL3	APC	DX27	Miltenyi Biotech	1/20
	KIR2DL5	APC	REA955	Miltenyi Biotech	1/100
**T-cell function**	Foxp3	PeCF594	236A/E7	BD Biosciences	1/100
**Viability**	Zombie	NIR		Biolegend	1/300

Supplementary Table 3: mAb list for CD8$^+$ T cells panel.

	Markers	Fluorochrome	Clone	Origin	Dilution
**Immune cell types**	CD3	AF532	UCHT1	Invitrogen	1/20
	CD4	BV510	RPA-T4	BioLegend	1/40
	CD8	BV570	RPA-T8	BioLegend	1/300
	CD56	APC-Cy7	HCD56	BioLegend	1/80
**Immune Activation / Maturation**	CD45RA	BV421	HI100	BioLegend	1/80
	CCR7	BV785	G043H7	BioLegend	1/20
	CD28	APC-R700	CD28 .2	BD Biosciences	1/80
	Hélios	PE-Dazzle594	22F6	BioLegend	1/40
**Immune checkpoint**	NKG2A	PE-Vio770	REA110	Miltenyi Biotech	1/400
	KIR2DL1	APC	REA284	Miltenyi Biotech	1/100
	KIR3DL1/DL2	APC	REA970	Miltenyi Biotech	1/100
	KIR2DL2/DL3	APC	DX27	Miltenyi Biotech	1/20
	KIR2DL4	APC	REA768	Miltenyi Biotech	1/100
	KIR2DL5	APC	REA955	Miltenyi Biotech	1/100
	NKG2C	VioBright	REA205	Miltenyi Biotech	1/100
	Nkp30	PE-Cy5	Z25	Beckman Coulter	1/20
	Nkp44	PE-Cy5	Z231	Beckman Coulter	1/20
	Nkp46	PE-Cy5	BAB281	Beckman Coulter	1/20
	PD1	BV650	EH12.2H7	BioLegend	1/40
**Viability**	zombie	NIR		BioLegend	1/300

Supplementary Table 4: mAb list for cytotoxic CD8$^+$ T cells panel

	Markers	Fluorochrome	Clone	Origin	Dilution
	CD3	AF532	UCHT1	Invitrogen	1/20
	CD4	BV510	RPA-T4	BioLegend	1/40
	CD8	BV570	RPA-T8	BioLegend	1/300
	CD56	APC-Cy7	HCD56	BioLegend	1/80
**CD8+ cytotoxic T-cells**	HLA1-a	BV711	pentamer	Proimmune	1/20
	HLA-E	PE	E*01 :01	Proimmune	1/20
**Immune Activation / Maturation**	CD45RA	BV421	HI100	BioLegend	1/80
	CCR7	BV785	G043H7	BioLegend	1/20
	CD28	APC-R700	CD28 .2	BD Biosciences	1/80
	Helios	PE-Dazzle594	22F6	BioLegend	1/40
	Nkp30	PE-Cy5	Z25	Beckman Coulter	1/20
	Nkp44	PE-Cy5	Z231	Beckman Coulter	1/20
	Nkp46	PE-Cy5	BAB281	Beckman Coulter	1/20
**Immune checkpoint**	NKG2A	PE-Vio770	REA110	Miltenyi Biotech	1/400
	KIR2DL1	APC	REA284	Miltenyi Biotech	1/100
	KIR3DL1/DL2	APC	REA970	Miltenyi Biotech	1/100
	KIR2DL2/DL3	APC	DX27	Miltenyi Biotech	1/20
	KIR2DL5	APC	REA955	Miltenyi Biotech	1/100
	NKG2C	VioBright	REA205	Miltenyi Biotech	1/100
	PD1	BV650	EH12.2H7	BioLegend	1/20
**Imune cell function**	GrzB/perf	PerCP-cy5.5	QA16A02/B-D48	Biolegend	1/100
**Viability**	zombie	NIR		BioLegend	1/300

Supplementary Table 5: IFNα and IFNλ paired-sera concentrations variations from individual patients collected before (UPs) and after (TPs) treatment.

		VL (particle/ml)		CD4 count (/ml)		INFα (fg/ml)		IFNλ (fg/ml)	
UPs	TPs	UPs	TPs	UPs	TPs	UPs	TPs	UPs	TPs
UP 4	TP 4	132773	<40	327	744	958.93	4.17	112328	115318
UP 11	TP 11	3012	<50	292	402	659.46	58.34	10424	9082
UP 3	TP 3	153719	NA	230	NA	441.17	0	371	197
UP 13	TP 13	<50	<50	639	603	161.74	7.62	17	114
UP 16	TP 16	11417	<40	740	1328	156.71	11.74	2868	4080
UP 6	TP 6	12015	<40	513	NA	138.01	0	65726	63032
UP 17	TP 17	63954	<40	344	410	128.63	12.39	1593	2166
UP 19	TP 19	17809	<50	196	437	127.82	5.07	451	365
UP 12	TP 12	29257	<40	477	627	119.62	13.48	2284	1192
UP 5	TP 5	1715	<40	419	NA	113.4	10.9	216	152
UP 2	TP 2	58059	<40	516	NA	90.02	0.01	2337	210
UP 14	TP 14	26059	<50	267	404	58.46	8.03	1139	10548
UP 18	TP 18	7180	<40	872	682	36.6	15.3	472	2227
UP 7	TP 7	21701	<40	447	NA	26.73	13.82	755	352
UP 10	TP 10	407	<50	445	503	26.1	68	12874	22199
UP 15	TP 15	5384	<50	470	459	23.76	17.79	244	19634
UP 9	TP 9	7011	<40	756	809	22.64	0	56	174
UP 1	TP 1	23808	<40	506	685	10.52	1.49	238	93
UP 8	TP 8	4785	<40	319	NA	5.65	1.63	1430	1627
HDs (med) n=65		none		NA		17.7		595	
UPs (med) n=19		14912		447		113		1139	
TPs (med) n=19		<40		603		8.03		1627	
ECs (med) n=18		114		878		15.27		672	

This table presents individual serologic levels of IFNα and IFNλ from HIV patients of the tested cohort, before (UPs) and after treatment (TPs), the viral loads and $CD4^{+}$ T-cell counts are also included.

The list is sorted by decreasing IFNα values in UPs. NA : Non Available; med : median

Supplementary Figure Legends

Supplementary Fig. 1: Gating strategy for immune cell types.

A- Gating strategy for immune cell types. The gating strategy used to identify the main cellular subsets is presented. Arrows are used to visualize the relationships across plots, and numbers are used to call attention to populations described here. After doublets and dead cells were excluded, lymphocytes were gated based on FSC$^-$A/SSC$^-$A properties. From the CD14$^-$CD19$^-$ lymphocyte gate, the following populations were identified: CD3$^+$TCRγδ$^+$, TCRγδ$^-$ were subdivided in CD3$^-$ and CD3$^+$ T-cells. NK cells were defined as CD3−TCRγδ−HLA$^-$DR−and classified as early NK (CD56$^+$CD16−), mature NK (CD56$^+$CD16$^+$), and terminal NK (CD56−CD16$^+$) cells. The CD3$^+$TCRγδ− population was divided in CD4$^+$ and CD8$^+$ T$^-$cells. In CD4$^+$ T$^-$cells subpopulation, CCR7$^+$ and CD45RA$^+$ were used to further classify these cells in four subpopulations: N (CCR7$^+$CD45RA$^+$), CM (CCR7$^+$CD45RA$^-$), EM (CCR7$^-$CD45RA$^-$) and TEMRA (CCR7$^-$CD45RA$^+$). Tregs were identified from the CD4$^+$ population using Foxp3 expression. Foxp3$^+$ cells were classified in naïve and memory Treg cells using CD45RA and CD25 markers. CD45RA$^-$CD25$^+$ represent the memory Treg cells population. As for CD4$^+$ T–cells, CD8$^+$ T–cells were classified using CD45RA and CCR7 markers: four populations were identified: N (CCR7$^+$CD45RA$^+$), CM (CCR7$^+$CD45RA$^-$), EM (CCR7$^-$CD45RA$^-$) and TEMRA (CCR7$^-$CD45RA$^+$). Among TEMRA CD8$^+$ T$^-$cells, we distinguished two cytotoxic subpopulations: iKIR$^+$ (CD8$^+$supp) and iKIR$^-$ (CTL). Dendritic cells (DCs) were identified by gating on CD3$^-$CD19$^-$CD56$^-$CD14$^-$HLA$^-$DR$^+$ and from there CD123$^+$CD11c$^-$ (pDCs) and CD11c$^+$CD123$^-$ mDCs were identified.

B- Specific markers analysed in each immune cell subsets.

Supplementary Fig. 2: Comparative immune phenotypic analysis of $CD4^+$ and $CD8^+$ T-cell subsets in UPs, TPs and in HDs.

(a) Boxplots showing the expression of indicated marker in $CD4^+$ naive **(a1)**, EM **(a2)** and TEMRA **(a3)** Tconv across the group (HDs n=22 (black), UPs n=10 (red) and TPs n=8 (blue)). **(a4)** Scatterplots showing relationships between the expression level of indicated markers in the $CD4^+$CM subsets (UPs n=10 and TPs n=8).

(b) Boxplots showing the expression of indicated marker in $CD8^+$ naive **(b1)**, EM **(b2)** and TEMRA **(b3)** populations across the group (HDs n=22, UPs n=10 and TPs n=8). **(b4)** Scatterplots showing relationships between the expression level of indicated markers in the $CD8^+$CM subsets (UPs n=10 and TPs n=8).

Multiple group comparisons were assessed through Kruskal–Wallis test with Dunn's multiple comparison testing, and correlation with Spearman's rank correlation test. Graph show the median values and p values (*P<0.05, **P<0.01, ***P<0.001, ****P<0.0001). ns: not significant. Error bars on graphs represent interquartile ranges.

Supplementary Fig. 3: TPs and ECs share few blood immune cell anomalies.

(a) Boxplots showing the expression of indicated marker in $CD4^+$ naive **(a1)**, CM **(a2)**, EM **(a3)** and TEMRA **(a4)** populations across the group : HDs (black), TPs (blue) and ECs (green) (**b)** Boxplots showing the expression of indicated marker in $CD8^+$ naive **(b1)**, CM **(b2)**, EM **(b3)** and TEMRA **(b4)** populations, and in TEMRA, CTL **(b5)** and $CD8^+$supp **(b6)** across the group (HDs n=22, ECs n=10 and TPs n=8). Multiple group comparisons were assessed through Kruskal–Wallis test with Dunn's multiple comparison testing. Graph show

the median values and p values (*P<0.05, **P<0.01, ***P<0.001, ****P<0.0001). ns: not significant. Error bars on graphs represent interquartile range

Supplementary Fig. 4: IFNα the key mediator of HIV pathogenesis.

Diagram shows the pathogenic mechanisms of HIV-1 and IFNα, resulting in a dysregulated immune response throughout the viral cycle. (**1**) HIV-1 and IFNα pathogenic vicious circles. (**2**) Functional inhibitory signaling checkpoint expression, such as PD-1, CD38, CTLA-4 or suppressive soluble mediators (IL-10) via immune suppression. (**3**) $EC_{\text{HLA-B57+}}$ cytotoxic NK-cells are not targeted by NK inhibitory killer iKIR, $EC_{\text{HLA-B57-}}$ NK cell cytotoxicity are stimulated via activating receptor NKG2C.

CPE: Cytopathic Effect; IR: Immune Reaction; * : Pathogenic IFNα = high IFNα levels;

**: scale of HIV pathogenesis;

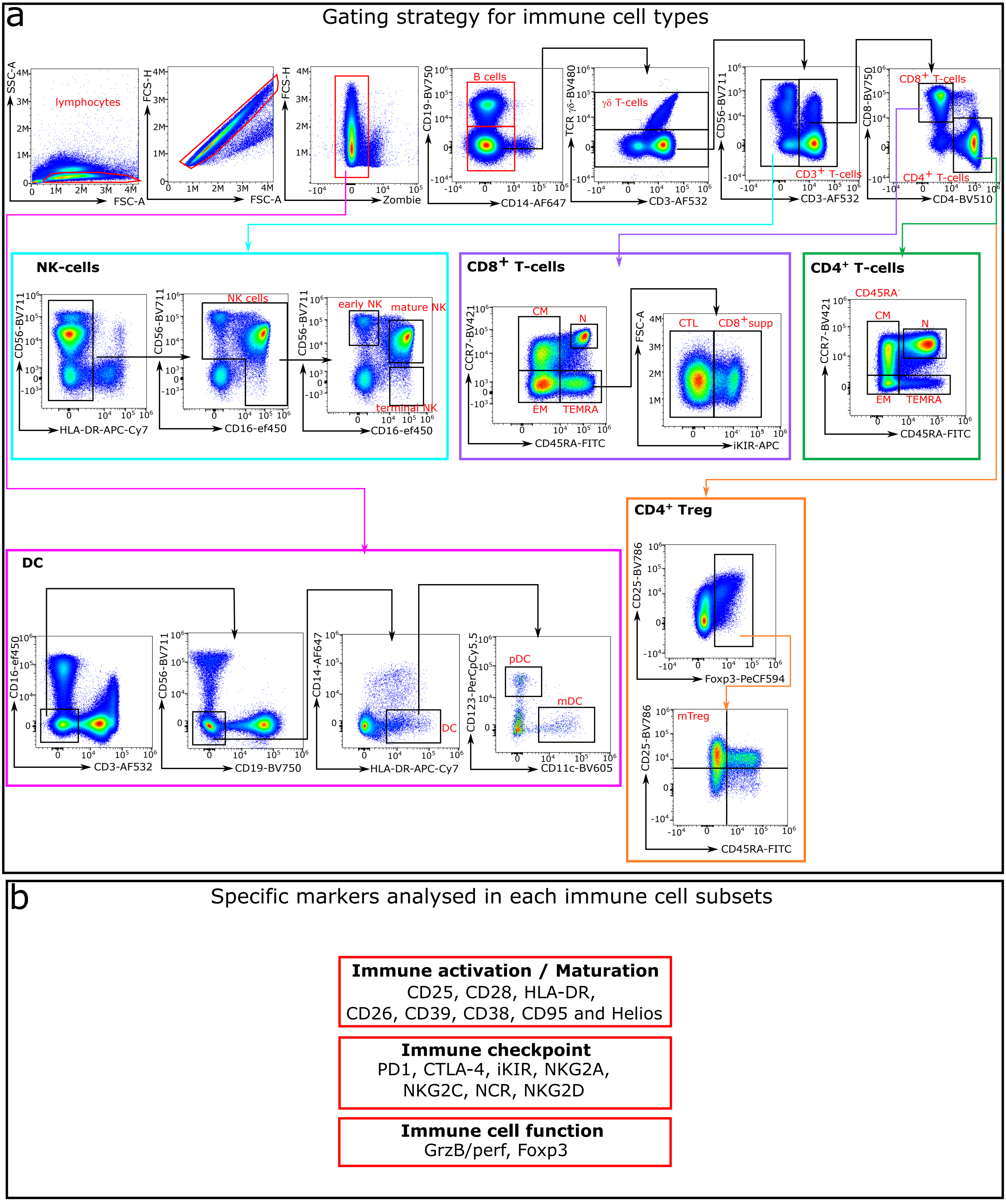

a
Gating strategy for immune cell types
lymphocytes
SSC-A
FSC-A
FSC-H
Zombie
B cells
CD19-BV750
CD14-AF647
γδ T-cells
TCR γδ-BV480
CD3-AF532
CD56-BV711
CD3+ T-cells
CD8+ T-cells
CD4+ T-cells
CD8-BV750
CD4-BV510
NK-cells
HLA-DR-APC-Cy7
NK cells
CD16-ef450
early NK
mature NK
terminal NK
CD8+ T-cells
CM
N
EM
TEMRA
CCR7-BV421
CD45RA-FITC
CTL
CD8+supp
FSC-A
iKIR-APC
CD4+ T-cells
CD45RA-
CD4+ Treg
CD25-BV786
Foxp3-PeCF594
mTreg
DC
CD16-ef450
CD19-BV750
CD14-AF647
CD123-PerCpCy5.5
pDC
mDC
CD11c-BV605
b
Specific markers analysed in each immune cell subsets
Immune activation / Maturation
CD25, CD28, HLA-DR,
CD26, CD39, CD38, CD95 and Helios
Immune checkpoint
PD1, CTLA-4, iKIR, NKG2A,
NKG2C, NCR, NKG2D
Immune cell function
GrzB/perf, Foxp3

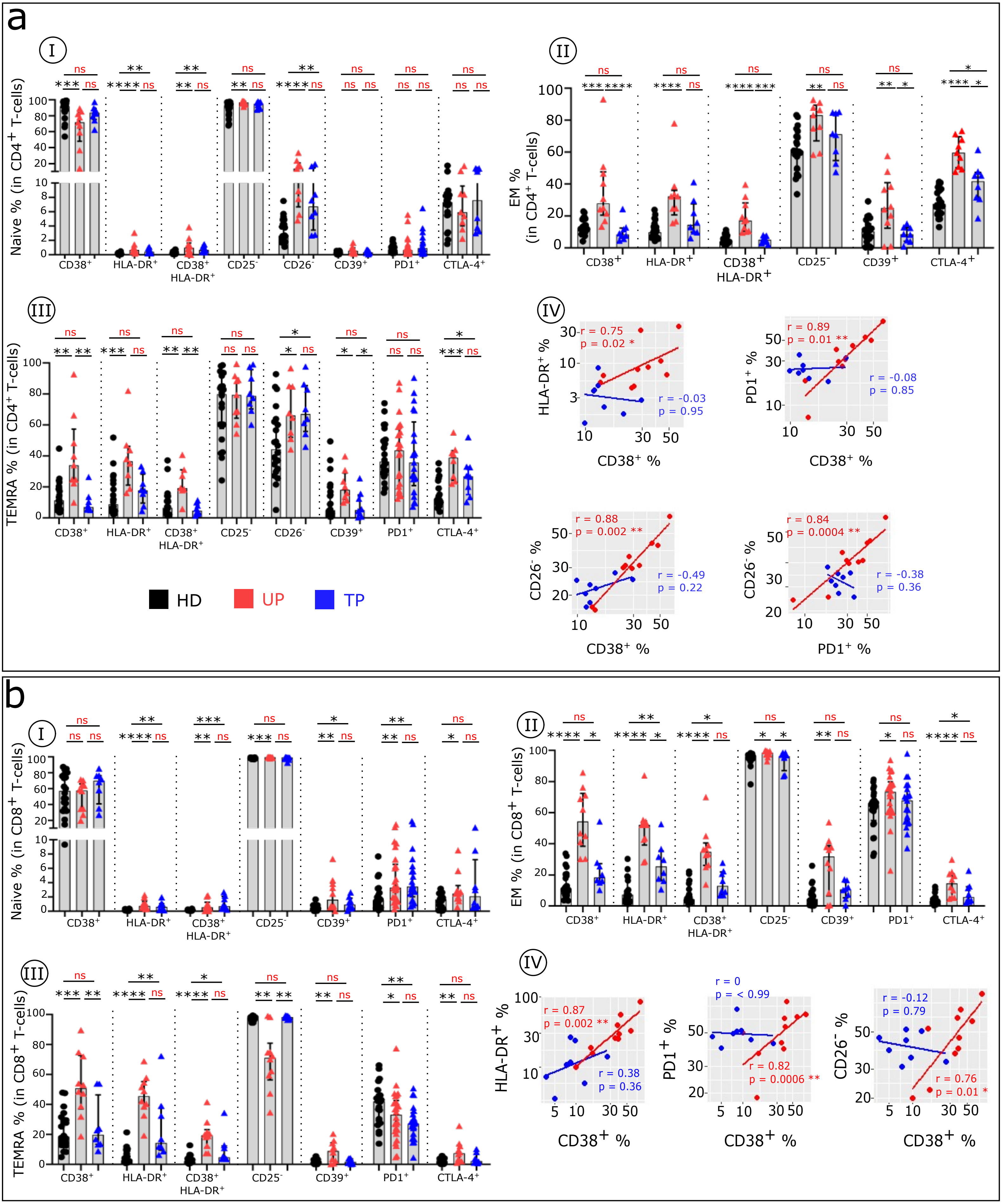
a
I
Naive % (in CD4+ T-cells)
CD38+
HLA-DR+
CD38+ HLA-DR+
CD25-
CD26-
CD39+
PD1+
CTLA-4+
II
EM % (in CD4+ T-cells)
III
TEMRA % (in CD4+ T-cells)
IV
HLA-DR+ %
PD1+ %
CD26- %
CD38+ %
PD1+ %
r = 0.75
p = 0.02 *
r = -0.03
p = 0.95
r = 0.89
p = 0.01 **
r = -0.08
p = 0.85
r = 0.88
p = 0.002 **
r = -0.49
p = 0.22
r = 0.84
p = 0.0004 **
r = -0.38
p = 0.36
HD
UP
TP
b
I
Naive % (in CD8+ T-cells)
II
EM % (in CD8+ T-cells)
III
TEMRA % (in CD8+ T-cells)
IV
HLA-DR+ %
PD1+ %
CD26- %
CD38+ %
r = 0.87
p = 0.002 **
r = 0.38
p = 0.36
r = 0
p = < 0.99
r = 0.82
p = 0.0006 **
r = -0.12
p = 0.79
r = 0.76
p = 0.01 *

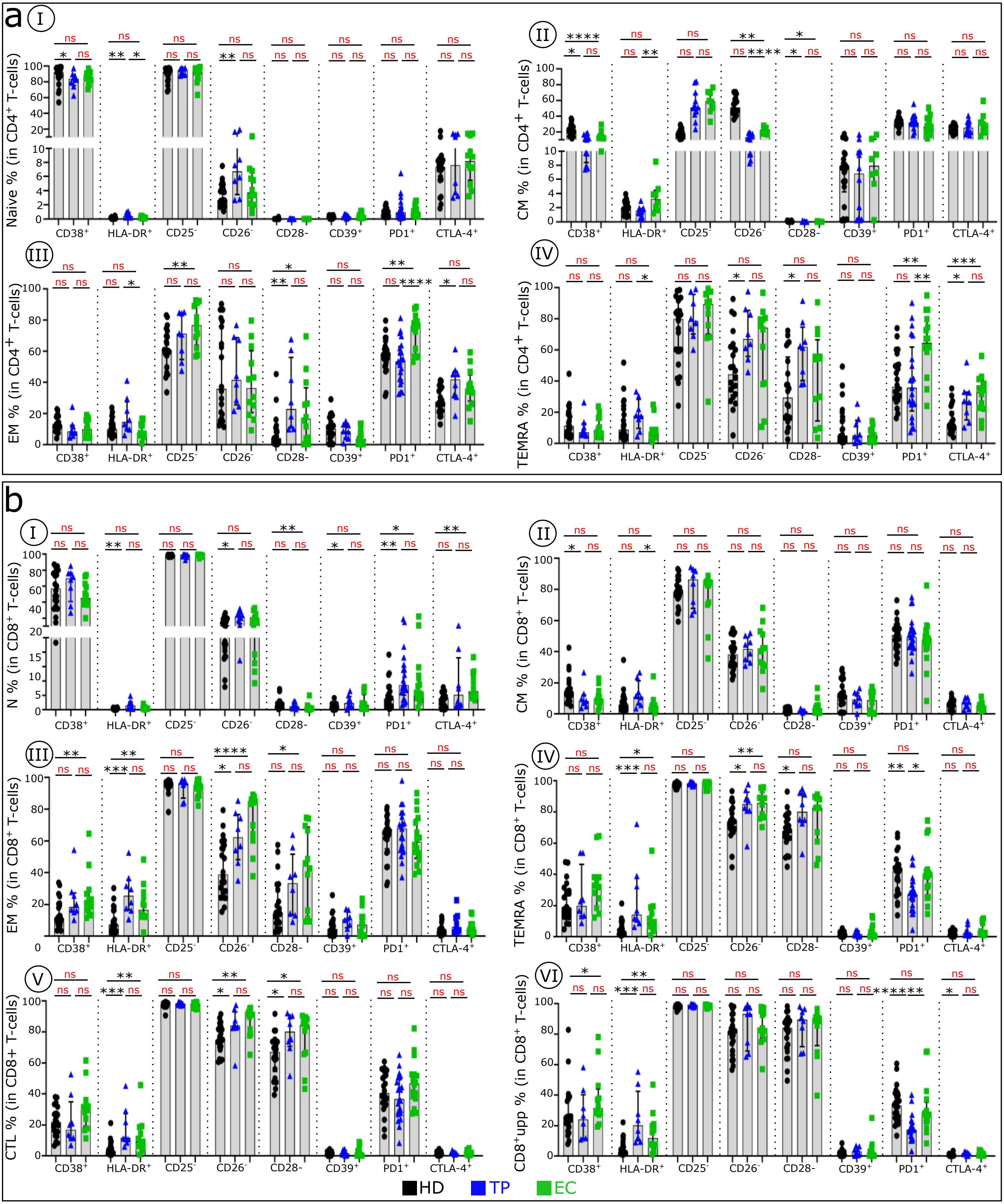

a
I
Naive % (in CD4+ T-cells)
II
CM % (in CD4+ T-cells)
III
EM % (in CD4+ T-cells)
IV
TEMRA % (in CD4+ T-cells)
CD38+
HLA-DR+
CD25-
CD26-
CD28-
CD39+
PD1+
CTLA-4+
b
I
N % (in CD8+ T-cells)
II
CM % (in CD8+ T-cells)
III
EM % (in CD8+ T-cells)
IV
TEMRA % (in CD8+ T-cells)
V
CTL % (in CD8+ T-cells)
VI
CD8+upp % (in CD8+ T-cells)
HD
TP
EC

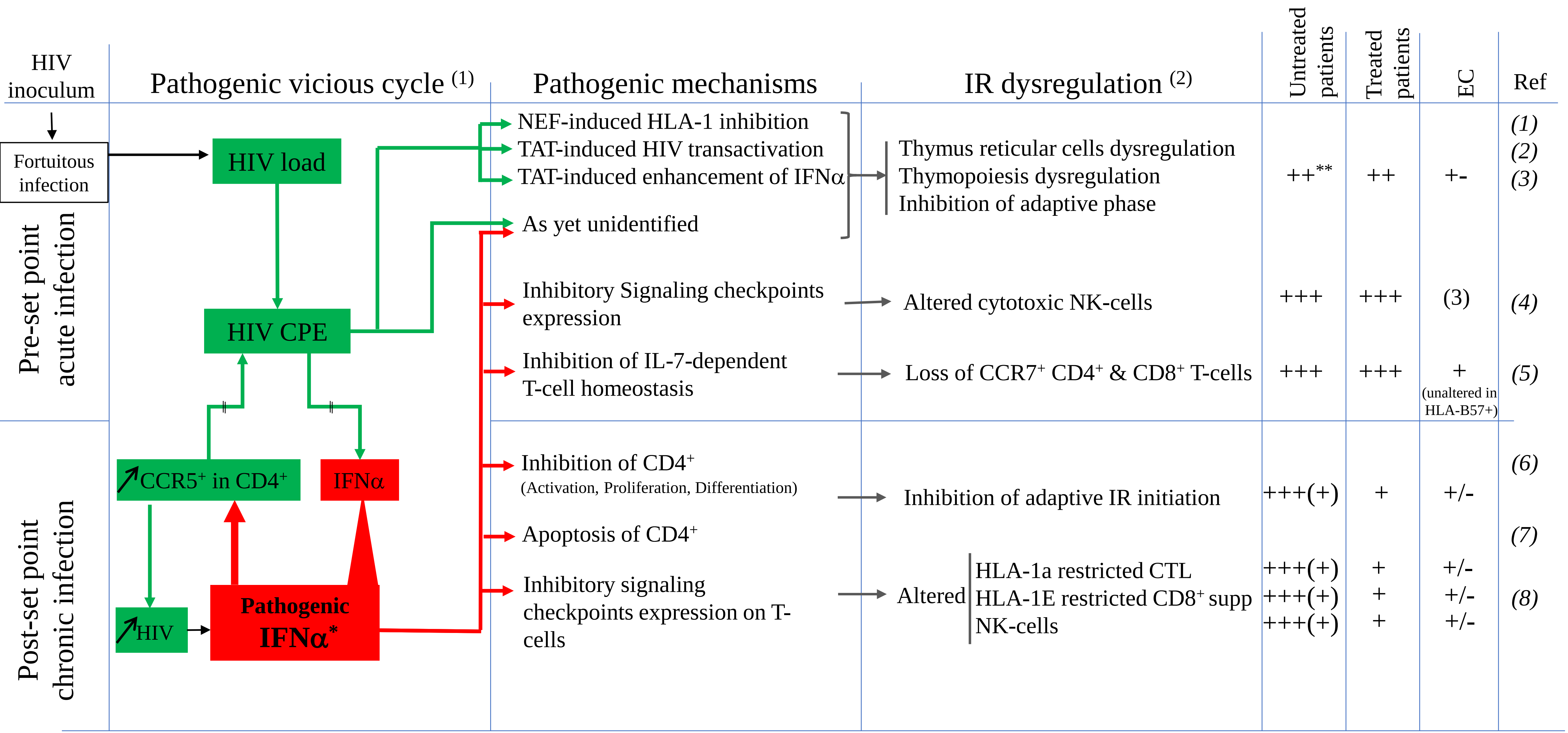

References :

1. Schwartz, O., Maréchal, V., Le Gall, S., Lemonnier, F. & Heard, J. M. Endocytosis of major histocompatibility complex class I molecules is induced by the HIV-1 Nef protein. Nat Med 2, 338–342 (1996).
2. Fisher, A. G. et al. The trans-activator gene of HTLV-III is essential for virus replication. Nature 320, 367–371 (1986).
3. Zagury, D. et al. Interferon alpha and Tat involvement in the immunosuppression of uninfected T cells and C-C chemokine decline in AIDS. Proc Natl Acad Sci U S A 95, 3851–3856 (1998).
4. Le Buanec, H et al. Early elevated IFNα is a key mediator of HIV pathogenesis. Comms Med. https://doi.org/10.1038/s43856-024-00454-6 (2024)
5. Cha, L., de Jong, E., French, M. A. & Fernandez, S. IFN-α Exerts Opposing Effects on Activation-Induced and IL-7–Induced Proliferation of T Cells That May Impair Homeostatic Maintenance of CD4 + T Cell Numbers in Treated HIV Infection. J.I. 193, 2178–2186 (2014).
6. Dondi, E., Rogge, L., Lutfalla, G., Uzé, G. & Pellegrini, S. Down-modulation of responses to type I IFN upon T cell activation. J Immunol 170, 749–756 (2003).
7. Herbeuval, J.-P. et al. Regulation of TNF-related apoptosis-inducing ligand on primary CD4+ T cells by HIV-1: role of type I IFN-producing plasmacytoid dendritic cells. Proc Natl Acad Sci U S A 102, 13974–13979 (2005).
8. Terawaki, S. et al. IFN-α directly promotes programmed cell death-1 transcription and limits the duration of T cell-mediated immunity. J Immunol 186, 2772–2779 (2011).
